# Akkermansia muciniphila modifies the association between metal exposure during pregnancy and depressive symptoms in late childhood

**DOI:** 10.21203/rs.3.rs-3922286/v1

**Published:** 2024-02-14

**Authors:** Vishal Midya, Kiran Nagdeo, Jamil Lane, Libni Torres-Olascoaga, Gabriela Martínez, Megan Horton, Chris Gennings, Martha Téllez-Rojo, Robert Wright, Manish Arora, Shoshannah Eggers

**Affiliations:** Icahn School of Medicine at Mount Sinai; Icahn School of Medicine at Mount Sinai; Icahn School of Medicine at Mount Sinai; Center for Research on Nutrition and Health, National Institute of Public Health; Center for Research on Nutrition and Health, National Institute of Public Health; Icahn School of Medicine at Mount Sinai; Icahn School of Medicine at Mount Sinai; Center for Research on Nutrition and Health, National Institute of Public Health; Icahn School of Medicine at Mount Sinai; Icahn School of Medicine at Mount Sinai; University of Iowa College of Public Health

## Abstract

Emerging research suggests that exposures to metals during pregnancy and gut microbiome (GM) disruptions are associated with depressive disorders in childhood. *Akkermansia muciniphila*, a GM bacteria, has been studied for its potential antidepressant effects. However, its role in the influence of prenatal metal exposures on depressive symptoms during childhood is unknown. Leveraging a well-characterized pediatric longitudinal birth cohort and its microbiome substudy (n=112) and using a state-of-the-art machine-learning model, we investigated whether the presence of *A.muciniphila* in GM of 9-11-year-olds modifies the associations between exposure to a specific group of metals (or metal-clique) during pregnancy and concurrent childhood depressive symptoms. Among children with no *A.muciniphila*, a metal-clique of Zinc-Chromium-Cobalt was strongly associated with increased depression score (*P*<0.0001), whereas, for children with *A.muciniphila*, this same metal-clique was weakly associated with decreased depression score(*P*<0.4). Our analysis provides the first exploratory evidence hypothesizing *A. muciniphila* as a probiotic intervention attenuating the effect of prenatal metal-exposures-associated depressive disorders in late childhood.

## Introduction

Depression is a global health burden, with 37% of adolescents experiencing elevated depressive symptoms worldwide between 2010 and 2020.^1^ Emerging research indicates that disruptions in gut microbiota and metabolites may contribute to the development of depressive disorders. ^2^ Likewise, beneficial or probiotic gut bacteria may help alleviate depressive symptoms. *Akkermansia muciniphila* is one such potentially beneficial bacteria that may be a promising avenue for preventive intervention of depression in childhood and adolescence. ^3^

*A. muciniphila* is a commensal inhabitant of the human gastrointestinal tract throughout life. ^3,4^ Extensive phylogenetic and metagenomic investigations have consistently identified *A. muciniphila* as one of the top 20 most prevalent species in the human gut. ^4–9^ Within the first year after birth, *A. muciniphila* can establish stable colonization in the gut, reaching levels similar to those found in healthy adults, with abundance gradually declining in the elderly. ^3,4,10^ *A. muciniphila* has also been detected in human milk, indicating its transfer from mothers to infants through breastfeeding. ^11^ This finding explains its presence in the gastrointestinal tract of newborn infants. ^3,4^ During this early developmental stage, *A. muciniphila* exhibits successful colonization in the gastrointestinal tract due to its active acid resistance system and capacity to degrade human milk oligosaccharides in the stomach of newborn infants.^3,12^ *A. muciniphila’s* inhabitance of the human gut microbiome (GM) during critical stages of human development may lead to *A. muciniphila* playing an important role in neurodevelopment via the gut-brain axis, which facilitates bidirectional interactions between the brain and the gut.^13^ The GM, including *A. muciniphila*, has been significantly associated with neuropsychiatric disorders, notably depression and anxiety.^14^ Using a mouse model of depression induced by chronic restraint stress (CRS), researchers found that *A. muciniphila* treatment significantly ameliorated depressive-like behavior in the CRS-exposed mice.^15^ Further, *A. muciniphila* supplementation could relieve depression-like symptoms and aggravation of colitis in recipient mice.^16^ These relationships may also exist in humans, though further epidemiologic investigation is needed.

Even at low levels, increased metal concentrations during childhood and adolescence have been associated with an increased risk of depression.^17,18^ Prenatal exposures to lead (Pb) and manganese (Mn) have also been associated with mid-childhood to adolescent internalizing symptoms.^19^ Exposure to multiple metals simultaneously may further strengthen this association. For instance, combined exposure to organochlorine and metal mixtures has been further associated with anxiety/depressive symptoms during adolescence. ^19^ While this evidence suggests a link between prenatal exposure to individual metals and some mixtures, not all components within a mixture are necessarily equally important.^20^ Having certain levels of exposure to specific combinations of few metals, or metal cliques, may have stronger associations than either individual metals or combinations of many metals.^21^ Metal cliques are a unique measure to assess associations because they lie in between individual components and mixtures of all components. However, few, if any, environmental epidemiology investigations have focused on metal cliques.

Exposure to toxic metals is also associated with the composition, diversity, and functional activities of the GM.^22–25^ We previously found that higher prenatal lead exposure reduced the diversity and abundance of beneficial taxa within the GM in children during mid-childhood (9-11 years)^26,27^ Although metals have been linked to both alterations of the GM and increased depression symptoms, the specific role of *A. muciniphila* in modifying the association between metal exposure and depression has yet to be examined. This study, therefore, investigates the role of *A. muciniphila* in modifying the association between prenatal metal clique and depressive symptoms in late childhood.

## Results

### Study Population

The study sample comes from the Programming Research in Obesity, Growth, Environment, and Social Stressors (PROGRESS) cohort in Mexico City. PROGRESS cohort is a well-characterized ongoing longitudinal birth cohort that has been continuously funded since 2007.^28^ Table 1 provides the descriptive statistics of the 112 participants included in this study, stratified by the presence/absence of *A. muciniphila* detected in 9-11-year-old gut samples. *A. muciniphilia* was present in 24% of participants. Mothers of children with *A. muciniphila* exhibited a lower BMI of 26.33 kg/m^2^ in pregnancy. The average t-scored Childhood Depression Inventory (CDI) was 52.97 (range: 40-81). On average, those without gut *A. muciniphila* had a higher CDI score (i.e., more depressive symptoms). For further analysis, we converted the relative abundance of *A. muciniphila* as a binary indicator of presence and absence to ward off any influence of outliers. The Spearman correlation between the metal concentrations is presented in Supplementary Figure 1. Details on concentrations of prenatal metals at both trimesters (with stratification by the presence of *A. muciniphila*) are shown in Supplemental Tables 1 and 2.

The p-values comparing PROGRESS participants with vs. without gut *A. muciniphila* were calculated using Fisher’s exact test or the Wilcoxon rank sum test, depending on the underlying variable.

### Individual Associations with Childhood Depressive Symptoms

**Figure 1:**
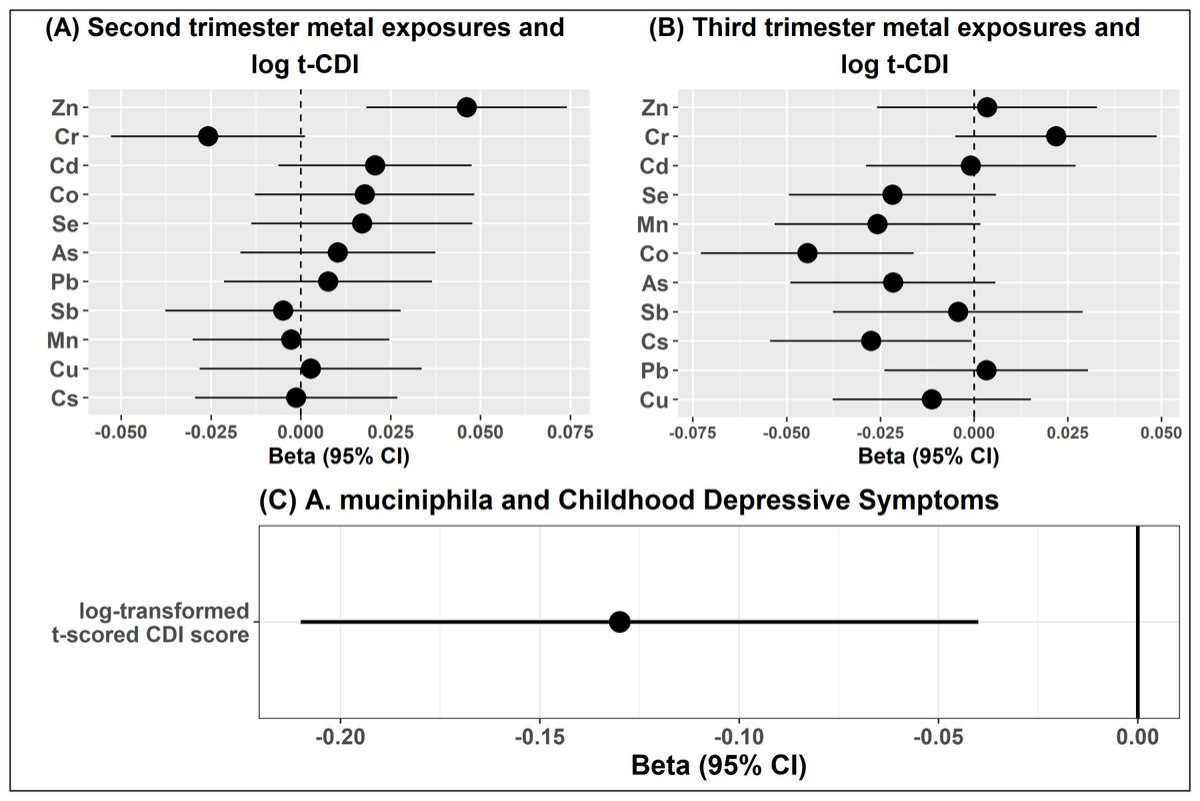
shows associations between individual metal exposures during pregnancy, the presence of *A. muciniphila* in childhood gut-microbiome, and log-tCDI scores among 112 PROGRESS children at 9-11 years of age. Figures 1A and 1B show associations (beta estimates and 95% CIs) with metal exposures in the 2^nd^ and 3^rd^ trimesters, respectively. The dotted vertical line denotes the null association. Figure 1C shows an association between the presence of *A. muciniphila* and Childhood Depressive Symptoms. t-CDI: t scored Child Depression Index.

We first presented the results of individual analyses with metals and the presence of *A. muciniphila*. The presence of *A. muciniphila* was associated with significantly decreased log-transformed, t-scored CDI (log-tCDI) (beta=−0.13, 95%CI=[−0.21,−0.04], randomization-based robust p-value (*P_rand_*<0.0001)) (Figure 1C). The result remained statistically significant and preserveed the directionality even on non-log transformed t-scored CDI. Metal concentrations of lead (Pb), arsenic (As), cadmium (Cd), chromium (Cr), zinc (Zn), selenium (Se), antimony (Sb), copper (Cu), cesium (Cs), cobalt (Co), and manganese (Mn) during the 2^nd^ and 3^rd^ trimester visits (18.3 and 31.6 weeks of gestation, respectively) were measured in maternal blood samples. A few individual metal exposures in the 2^nd^ and 3^rd^ trimesters of pregnancy were significantly associated with log-tCDI at 9-11 years of age (Figure 1A and 1B). In the 2^nd^ trimester, higher Cr exposure was associated with decreased log(t-CDI) in late childhood (β[95% CI]= −0.03[−0.05, 0.00],*P*=0.05), whereas higher Zn exposure was associated with an increase in log(t-CDI) score (β[95% CI]=0.04[0.01, 0.06], *P*=0.008). Similarly, in the 3^rd^ trimester, higher Co and As were associated with decreased log(t-CDI) scores (β[95% CI]=−0.04[−0.07, −0.02], *P*=0.002 and −0.03[−0.06, −0.01], *P*=0.02, respectively), while Cr was associated with increased log(t-CDI) (β[95% CI]=0.03[0.00, 0.05], *P*=0.04). Only the false discovery rate (FDR)-adjusted p-value associated with Co, in the 3^rd^ trimester, was lower than 0.05.

### Prenatal metal-clique, *A. muciniphila*, and Childhood Depressive Symptoms

We used an interpretable machine learning model called repeated holdout signed-iterated Random Forest (rh-SiRF),^27,29,30^ along with a regression framework to identify metal cliques that are both predictive and associated with depression score (see Methods). We obtained a total of 123 unique two-component cliques from rh-SiRF, with only 3% of cliques having stability (frequency of occurrence) of more than 5% (see Supplemental Table 3 for the list of top 10 combinations). We chose the top three combinations since those formed a closed-looped network (Supplementary Figure 2 for a forced-directed graph of this clique). We identified a three-component metal clique composed of (1) high Zn in the 2^nd^ trimester (concentration greater than 20^th^ percentile of the sample), (2) low Co in the 3^rd^ trimester (concentration below 80^th^ percentile of the sample), (3) low Cr in the 2^nd^ trimester (concentration below 55^th^ percentile of the sample). This three-component metal-clique is essentially a binary indicator that specifies a subgroup of children (comprising almost 40.9% of the sample) using the interactions of a few metals. Next, this clique was used in a regression framework to estimate the associations with (log-tCDI scores. We used a matched-sampling strategy typically applied in causal inference analysis to obtain similar covariate distribution between children with or without A for improved inference. *muciniphila*.^31^ The assumption is that, given the covariates, this balancing approach can potentially create “exchangeable” groups of children with or without *A. muciniphila* such that they are hypothetically randomly assigned, and most importantly, the covariates did not confound the group assignment.^32^ We conducted the regression analysis on the covariate-balanced dataset, further controlling for covariates and confounders.

**Figure 2:**
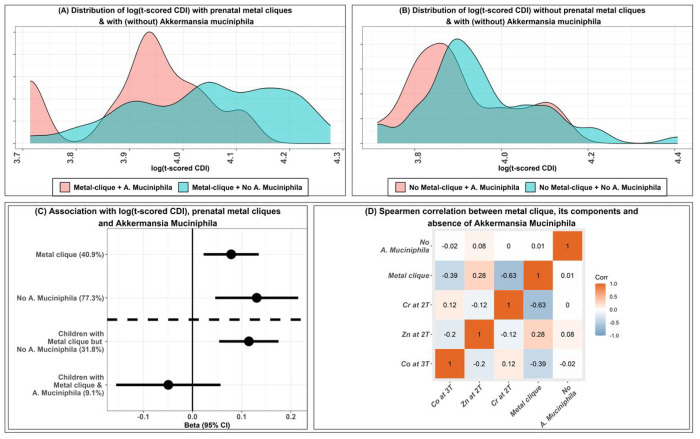
shows the distribution and the association with prenatal metal-clique and log-tCDI scores and the effect modification by the absence of *A. muciniphila* in childhood gut microbiome. Figure 2A shows the distribution of log-tCDI scores for children with the metal-clique (and with/without *A. muciniphila*). Figure 2B shows the distribution of log-tCDI scores for children without the metal-clique (and with/without A. muciniphila). Figure 2C shows the beta coefficients and 95% CIs for the association with prenatal metal-clique and log-tCDI scores and the effect modification by the absence of *A. muciniphila*; the proportions of the sample characterized by the clique are shown in brackets on the y-axis. Figure 2D shows the Spearman correlations among the components of the metal-clique (individual metal concentrations), the metal-clique, and the indicator for the absence of *A. muciniphila* among the 112 PROGRESS children. t-CDI, t scored Child Depression Index.

The distributions of the log-tCDI were shown in Figures 2A and 2B for children with (and without) the metal clique and with (without) *A. muciniphila*. Children without the metal clique (and with/without *A. muciniphila*) have a similar distribution of log-tCDI. In contrast, the distributions of children with the metal clique differ drastically with or without *A. muciniphila*. The three-component metal-clique of high Zn, low Cr in the 2^nd^ trimester, and low Co in the 3^rd^ trimester was significantly associated with an increased depression score (β[95% CI]=0.08[0.02, 0.13], *P_rand_*<0.0001) (Figures 2C). However, this metal-clique was not correlated with the presence of *A. muciniphila* (Figure 2D), indicating that *A. muciniphila* is not a mediator between this metal-clique and childhood depression scores. However, the presence of *A. muciniphila* modifies this association. For 31.8% of children with no *A. muciniphila*, the metal-clique was strongly associated with increased depression score (β[95% CI]=0.11[0.05, 0.18], *P_rand_*<0.0001), whereas, for children with *A. muciniphila*, this same metal-clique was weakly associated with decreased depression score in almost 9.1% children, although the association was not statistically significant (β[95% CI]=−0.05[−0.16, 0.06], *P_rand_*<0.4). We further estimated the Spearman correlations between the components of this metal-clique, the overall indicator of the three-component metal-clique, and the absence of *A. muciniphila* (Figure 2B). Correlations between the metal components were minimal, which implies that correlation between metal concentrations did not have a significant effect in forming the clique, indicating a possibility of non-linear interaction.

### Sensitivity Analyses

We conducted multiple sensitivity analyses to substantiate our results: (1) For the major associations (except the forest plots), we estimated randomization-based p-values (*P_rand_*) (that are robust against any assumption of normality) by permuting each of the outcomes 10^6^ times. The significant *P_rand_* values were far lower than the model-based p-values (Supplemental Table 4). (2) Given the small sample size, we chose a minimal set of covariates to adjust in the models but incorporated techniques like covariate-balancing to obtain robust results (the covariate-balanced love plot is presented in Supplemental Figure 3). (3) Moreover, the presence of *A. muciniphila* remained strongly associated with a significantly decreased log-tCDI scores even on the non-covariate balanced dataset (beta=−0.10, 95%CI=[−0.18, −0.01]). (4) The directionalities of all the metal-clique associations (with and without the presence of *A. muciniphila*) remained unaltered while each of the thresholds was increased and decreased by ten percentiles (Supplemental Figures 4 and 5), implying greater robustness. (5) Results remained robustly similar when we binarized the t-scored CDI (>= 75^th^ percentile) and repeated the metal-clique associations (Supplemental Figures 6). (6) We used a negative control outcome – having a pet (at the time of microbial sample collection), which might have similar sources of potential selection bias but would not be causally related to the identified metal-clique. In the association with metal-clique, we found a null association with different directionality (OR[95% CI]:0.7[0.2,2.5]) between metal-clique and having a pet, strengthening the possibility of minimal selection and residual confounding biases.

## Discussion

In this study, we explored the modifying effect of *A. muciniphila* on the associations between prenatal exposure to a metal-clique and depressive symptoms in late childhood. Our results suggest that children with exposure to metal-clique of Zinc-Chromium-Cobalt during pregnancy had a higher CDI score in late childhood. In the absence of *A. muciniphila* in childhood GM, this metal-clique was strongly associated with higher depression symptoms in children. However, for children with *A. muciniphila*, this metal-clique was weakly associated with lower depression symptoms. This analysis provides the first exploratory evidence that the presence of *A. muciniphila* likely attenuates the association between prenatal exposure to metals and depression in later childhood.

*A. muciniphila* is a symbiotic bacterium colonizing the intestinal epithelium’s mucosal layer. This mucus layer comprises high-molecular-weight glycoproteins called mucins.^33^ *A. muciniphila* breaks down mucins, producing short-chain fatty acids (SCFAs) that facilitate the bacteria’s colonization process and provide energy and neuromodulators for the host.^34^ These SCFAs contribute to the maturation of the immune and neurological systems. ^34^ As a member of the GM, *A. muciniphila* communicates with the central nervous system (CNS) via the microbiota-gut-brain axis (MGBA). ^35–37^ Reduced *A. muciniphila* levels have been seen in mice and rats displaying depression-like behavior.^38,39^ In the FinnBrain Birth Cohort, the prevalence of *Akkermansia* was inversely associated with maternal postpartum depression symptoms.^40^ Depression is associated with lower levels of brain-derived neurotrophic factor (BDNF), and the neurotrophic hypothesis connects this decrease in BDNF to the pathophysiology of depression. ^41–43^ *A. muciniphila* counters this by enhancing BDNF expression, fostering synaptic pathways, and reducing depression symptoms.^2,14,15^ Individuals with depression often have reduced 5-HT (5-hydroxytryptamine or serotonin) levels, aligned with the monoamine deficiency hypothesis, which posits that the fundamental physiological cause of depression involves a reduction in serotonin, norepinephrine, and/or dopamine within the CNS. ^44–46^ *A. muciniphila* positively influences host 5-HT levels in the intestine through factors like its outer membrane protein Amuc_1100, which elevates intestinal 5-HT expression.^47,48^ When considering our findings with those presented from these animal and human studies, there appears to be a consistent negative correlation between the presence and prevalence of *Akkermansia* and depressive behavior, with multiple potential biological mechanisms.^14^ This recurring pattern strongly suggests the potential utility of *Akkermansia* as a viable strategy to alleviate depression symptoms; however, further longitudinal intervention studies with large samples are needed. ^14^

We found that presence of a prenatal metal-clique, including high Zn and low Cr in the second trimester and low Co in the third trimester, was associated with a higher depression index in children, and the association was reduced in children who had *A. muciniphila* in the GM. Previous research has indicated that heavy metal exposure can initiate neuro-inflammation, oxidative stress, hormonal imbalances, and disruptions to neurotransmitters like dopamine and serotonin, all of which can contribute to the etiology of depressive disorders. ^49–52^ For example, Rokoff et al. show that prenatal exposure to Pb was found to be linked to increased anxiety symptoms during adolescence. In contrast, prenatal exposure to Mn was positively correlated with internalizing symptoms, particularly among girls from mid-childhood through adolescence. ^19^ Gari et al. report that prenatal concentrations of micronutrients, Se and Zn, and neurotoxic metals, Pb and Hg, exert notable influences on the neuropsychological development of children at the age of 7.^53^ A previous study by our group found that a metal-microbial clique of high Zn in the second trimester, low Co in the third trimester, and high abundance of *Bacteroides fragilis* and *Faecalibacterium prauznitzii* in childhood was associated with increased depression scores.^21^ Co is a component of Vitamin B12, which may be associated with depression,^54,55^ and has been previously associated with *A. muciniphila*.^56^ The results of our study in the context of these previous findings suggest that in-utero exposure to metals could be particularly important in contributing to the development of anxiety and depression symptoms.

Exposure to metals can impact GM composition and function, especially during early development. The existing body of knowledge about the interactions between metals and *Akkermansia* in human physiology is limited, and exposures during the prenatal period appear entirely unexplored with *Akkermansia*. Our analysis did not indicate strong correlations between the prenatal metal-clique and *A. muciniphila*; however, previous research indicated that toxic metals like Cd, Pb, Cu, and Al reduced *A. muciniphila* levels in mice and common carp. ^57–61^ Shen et al., found that higher childhood blood Mn may lead to lower mucin degradation and energy generation and is significantly associated with lower *Akkermansiaceae*. ^23^ Their findings indicate a potential association between metal exposure in childhood and *Akkermansia* abundance, but they did not find an association with earlier exposures. Although there is limited evidence of interaction between *Akkermansia* in GM and metal exposure, there is evidence of potential biological mechanisms that may combine to influence human health. Metal exposure may alter the relative abundance of *Akkermansia* within the GM or reduce its ability to produce SCFAs,^62,63^ either of which may reduce *Akkermansia*’s ability to communicate with the CNS and potentially influence depression etiology. *A. muciniphila* also helps strengthen the epithelial barrier in the gut, ^64^ while metal exposures can negatively impact gut barrier function.^65^ Our finding of modification by *A. muciniphila* between metal cliques and depression may function through improved epithelial barrier strength. Alternative mechanisms may also exist, supporting the need for further investigation in this area.

While this study contributes to the growing body of evidence concerning the adverse link between metal exposure, depression, and human GM, some limitations must be acknowledged. The sample size limited our ability to make more robust conclusions due to a lack of power. Nevertheless, consistent associations across various sensitivity analyses and using causal-inference methods boost the inferences, even though some estimates did not reach statistical significance. Additionally, measuring prenatal metal exposure through maternal blood during pregnancy is suboptimal as it does not directly gauge fetal metal exposure. In the analysis, we did not control the models by any diet-related covariates due to a lack of information collected during the survey. Strengths of this study include the novel investigation of *Akkermansia* as a modifier of prenatal metal exposures and childhood depression. We performed robust statistical analysis, applying tools from current state-of-the-art machine-learning and causal inference literatures and using the presence of *A. muciniphila* instead of its relative abundance to minimize plausibility of measurement error. Our analysis of metal-cliques adds novel insight into combinations of metal exposures that are found in susceptible subgroups of the population, potentially making them more vulnerable to childhood depression. Findings from this study have translational potential, indicating *A. muciniphila* as an intervention avenue to help prevent depression in children with prenatal metal exposures. Additional future directions of this work include mediation analysis with depression measured later in adolescence and *in vitro* and animal experiments to help establish the biological plausibility of associations between metal exposures and *A. muciniphila* and mechanisms of modification.

This study suggests that the presence of *A. muciniphila* may attenuate the effect of prenatal exposure to a select metal-clique on childhood depression. Further observational, experimental, and translational investigation is needed to fully understand the occurrence, mechanisms, and potential interventions along this pathway.

## Methods

### Study Population

PROGRESS is an ongoing prospective birth cohort study conducted in Mexico City, Mexico. The study enrolled 948 women affiliated with the Mexican Social Security Institute (IMSS) during early pregnancy, and closely tracked the development of the infants during their early years, with assessments every six months initially and later biannually. Comprehensive longitudinal follow-up included surveys, physical examinations, and psychological/behavioral evaluations. Biological samples from mothers and children, including blood, were collected and archived at each visit. Additionally, a convenience sampled subset of participants (n = 123) contributed stool samples when the children reached ages 9–11 years.^26^ Of the 123 participants with stool samples, 112 also had complete outcome data. The research protocols for both the main PROGRESS study and its microbiome sub-study underwent thorough review and were granted approval by the Institutional Review Board at the Icahn School of Medicine at Mount Sinai in New York and the National Institute of Public Health in Cuernavaca, Mexico.

### Blood metal measurement

During the 2^nd^ and 3^rd^ trimester visits (18.3 and 31.6 weeks of gestation, respectively), maternal blood samples were collected using standard venipuncture. ^66^ All blood specimens were drawn using tubes free from trace metals and were stored at temperatures between 2°C and 6°C until analysis. Metal concentrations, including lead (Pb), arsenic (As), cadmium (Cd), chromium (Cr), zinc (Zn), selenium (Se), antimony (Sb), copper (Cu), cesium (Cs), cobalt (Co), and manganese (Mn), were determined using the Agilent 8800 ICP Triple Quad (ICP-QQQ) in MS/MS mode at the trace metals laboratory of the Icahn School of Medicine at Mount Sinai. Measurements were taken in five replicates and reported as an average. For the purpose of QC, all lab recovery rates by this method were 90 to 110%, and inter-day and intra-day precision (given as %relative standard deviation) is less than 6% for samples with concentrations greater than the limit of quantification. ^67^ The limits of detection for each metal were: 0.391 ug/L for As, 0.113 ug/L for Cd, 0.117 ug/L for Co, 0.901 ug/L for Cr, 0.122 ug/L for Cs, 1.862 ug/L for Cu, 0.442 ug/L for Mn, 0.377 ug/L for Pb, 0.159 ug/L for Sb, 0.665 ug/L for Se, and 5.053 ug/L for Zn (See Midya, 2024 for more details).^21^

### *Akkermansia muciniphila* measurement

Details of the gut microbiome sampling and processing procedures have been previously published.^26^ Briefly, participants were recruited during the 9–11-year PROGRESS study visit. Stool samples were collected by participants at home, processed using the Fast method at the clinic in Mexico City, and promptly stored −70°C. Frozen samples were then shipped to the Microbiome Translational Center at Mount Sinai for microbiome sequencing analysis. The subsequent steps involved the processing and sequencing of the samples in two distinct batches: the first batch containing 50 samples and the 2^nd^ containing 73 samples. For shotgun metagenomic sequencing, the NEBNext DNA Library Prep kit was used, and purified DNA sequencing using the Illumina HiSeq platform. The sequencing reads underwent trimming using Trimmomatic.^68^ To eliminate any human-associated reads, alignment to a human reference genome was conducted using bowtie2. ^69^ The remaining reads underwent analysis with MetaPhlAn2 and StrainPhlAn to ascertain detailed microbial taxonomy down to the species and strain levels.^70,71^ Results of the sequencing process can be found in Eggers, et al, 2023. Only children with no antibiotic use within the past month were included in this sub-study. For this analysis, we used the presence/absence of *A. muciniphila* from this sequencing data.

### Depressive Symptom Measurement

As a part of the neurobehavioral follow-up, childhood depressive symptoms were assessed using the Childhood Depression Inventory (CDI) 2 Self-Report Short Version at ages 9-11 years.^72^ Part of the CDI form was administered to mothers about their children, and part of the form was administered directly to the children. The CDI is a questionnaire appropriate for use with participants aged 7-17 years and validated in Spanish.^73^ Items are scored on a scale normalized from 0 to 100, with higher scores indicating worse depression symptoms.

### Covariate data

We followed a similar set of minimal covariates in line with our previous work. ^27,29,30^ Potential confounding variables used in this analysis encompassed a range of factors identified in previous literature and prioritized using DAGs. These included the child’s reported sex at birth, age at the time of stool sample collection, the socioeconomic status (SES) of the mother during pregnancy, the mother’s age at childbirth, the mother’s 2^nd^-trimester body mass index (BMI) while pregnant, and the batch of microbiome analysis (two batches). The mother’s height and weight were measured during the 2^nd^ trimester using a professional digital scale and a stadiometer. From these measurements, the BMI was calculated and subsequently treated as a continuous covariate for regression analyses. The 1994 Mexican Association of Intelligence Agencies Market and Opinion (AMAI) rule was employed to assess maternal socioeconomic status during pregnancy. This classification system places families into six levels based on responses to 13 questions concerning household characteristics. As most families in the study belonged to the low to middle SES bracket, these six categories were consolidated into three broader classifications: lower, middle, and higher.^74^ All of these covariates were adjusted for in the statistical models. This sub-study utilized convenience sampling due to proximity to study location, availability of mothers and their children at a given time, or their willingness to participate in the study. Although there is potential selection bias and residual confounding bias, we utilized advanced causal inference techniques to address such issues to the best of our ability. Our analyses utilized covariate-balancing techniques and negative control outcomes to address any systematic and potential selection and residual confounding biases.

### Statistical Analysis

Statistical analyses were conducted in R (version 4.2.3). The Pearson correlation coefficient was used to estimate the correlation between prenatal metal exposures. The outcome, t-scored CDI, was log-transformed (log tCDI) for all the main analyses (later in the sensitivity analyses, similar results were replicated with the non-log-transformed t-scored CDI). Some covariates (mother’s age and child’s age at the time of stool collection) included less than 5% missing values; therefore, under the assumption of missing at random, we imputed the missing values using predictive mean matching by the multiple imputation chained equations as implemented in the “MICE” R package.^75^ A false discovery rate was used to adjust for multiple comparison errors. The statistical analysis was conducted in three stages. First, we estimated the association with each individual metal and log-tCDI using linear regression models. The results were presented through a forest plot with beta estimates and corresponding 95%CI. Second, we estimated the association between the presence of *A. muciniphila* and log-tCDI using linear regression. Third, using a combination of interpretable machine-learning algorithm and regression-based framework, we identified the most frequently occurring metal-cliques and then estimated their associations with log-tCDI.

Although the details can be found in previous works, we present and discuss this method comprehensively for interpretability and further replicability.^27,29,30^ A metal-clique is an indicator of a multi-ordered combination of metals. For example, consider a three-metal combination consisting of metals A, B, and C, denoted as A+B+C−. Here, the metal-clique A+B+C− implies higher concentrations of metals A and B (above certain thresholds) and a lower concentration of metal C in the sample. This binarized indicator form of metal-clique A+B+C− denotes an underlying sub-sample (or subgroup) satisfying the conditions of the clique. We used the repeated holdout signed-iterated Random Forest (rh-SiRF),^27,29,30^ treating the concentrations of metal exposures during both the second and third trimesters as predictors and log(t-CDI) as the outcome. The rh-SiRF algorithm utilizes a combination of “Iterative Random Forests” with “Random Intersection Trees” to search for metal combinations predictive of log(t-CDI) scores. ^76–78^These predictive combinations of metals were chosen following the branches in the decision trees. Moreover, instead of searching for all possible metal combinations (231 two-components, 1540 three-components, for example), rh-SiRF finds the most frequently occurring combinations on the decision path. Further, bagging and repeated-holdout stages were introduced to estimate the “stability” of the discovered combinations. The algorithm was repeated 500 times on a training/test data partitioning of 60%/40%, with 250 bootstraps implemented in each iteration. From the list of most stable metal combinations, we chose the top three. We presented a closed-loop network of this combination through the Fruchterman-Reingold Layout ^79^ implemented through the igraph package in R.^80,81^ Finally, this combination is transformed into a binarized indicator using a quantile-based threshold-finding algorithm. A schematic of this algorithm and R code with illustrations on a simulated dataset is provided on GitHub (https://github.com/vishalmidya/MiCA-Microbial-Co-occurrence Analysis/blob/main/MiCA-vignette.md).

For improved inference, we used a matched-sampling strategy typically applied in causal inference analysis to obtain similar covariate distribution between children with or without *A. muciniphila*.^31^ The assumption is that, given the covariates, this balancing approach can potentially create “exchangeable” groups of children with or without *A. muciniphila* such that they are hypothetically randomly assigned, and most importantly, the covariates did not confound the group assignment.^32^ Due to the small sample size and to prevent greater sample loss due to covariate-balancing, we used a subclass matching procedure with the propensity score as implemented in the R package “MatchIt”.^82^ This approach uses propensity scores based on all the covariates to classify participants into subclasses, which were then weighted to balance the influence of the covariates for participants with vs. without *A. muciniphila*. We used love plots of the differences in standardized means in covariates to examine the suitability of balancing.^32^ All the regression analyses were based on this covariate-balanced matched sample. We further adjusted all the models with the previously described covariates.

## Figures and Tables

**Table 1.** Descriptive statistics of covariates, and depression scores from 9-11 years of age PROGRESS participants included in this study (n = 112).

	Overall	A. muciniphila Absent	A. muciniphila Present	p-value
*Covariates*				
Child Sex				0.83
Male *n(%)*	68 (60.7)	51 (60.00)	17 (63.0)	
Female *n(%)*	44 (39.3)	34 (40.00)	10 (37.0)	
Maternal SES in pregnancy				0.71
Lower *n(%)*	61 (54.5)	48 (56.5)	13 (48.2)	
Medium *n(%)*	41 (36.6)	29 (34.1)	12 (44.4)	
Higher *n(%)*	10 (8.9)	8 (9.4)	2 (7.4)	
Maternal age at birth (years) *mean(Sd)*	28.7 (5.8)	29.2 (5.9)	27.1 (5.4)	0.12
Maternal BMI in pregnancy (kg/m^2^) *mean(Sd)*	27.3 (4.5)	27.6 (4.6)	26.3 (4.2)	0.28
Child age at gut microbial sample collection (years) *mean (Sd)*	9.7 (0.9)	9.6 (0.9)	9.8 (1.0)	0.42
*Outcome*				
t-scored Childhood Depression Inventory *mean(Sd)*	53.0 (8.1)	53.7 (8.1)	50.7 (7.8)	0.07

## Data Availability

Metagenomic data are publicly available at https://www.ncbi.nlm.nih.gov/sra/PRJNA975184. All other data are available upon request to robert.wright@mssm.edu.

## Abbreviations

A. muciniphila: Akkermansia muciniphila
Pb: Lead
As: Arsenic
Cd: Cadmium
Cr: Chromium
Zn: Zinc
Se: Selenium
Sb: Antimony
Cu: Copper
Cs: Cesium
Co: Cobalt
Mn: Manganese
WQS: Weighted Quantile Sum
log tCDI: Logarithm transformed t-score normalized Children’s Depression Inventory
PROGRESS: Programming Research in Obesity, Growth, Environment, and Social Stressors
IQR: Inter Quartile Range
